# How do health care services help and hinder recovery after a suicide attempt? A qualitative analysis of Finnish service user perspectives

**DOI:** 10.1186/s13033-022-00563-6

**Published:** 2022-11-16

**Authors:** Selma Gaily-Luoma, Jukka Valkonen, Juha Holma, Aarno Laitila

**Affiliations:** 1 University of Jyväskylä, Jyväskylä, Finland; 2 MIELI Mental Health Finland, Helsinki, Finland

**Keywords:** Suicide attempt, Self-harm, Health care, Service user, Experience, Mental health, Psychiatry, Emergency services, Qualitative, Recovery

## Abstract

**Background:**

Suicide attempt survivors are at high risk of re-attempts and suicide death. Previous research has shown that service users’ experiences of post-attempt care are related to future treatment engagement and re-attempts. In-depth understanding of how current services meet service users’ needs in the period immediately following a suicide attempt is thus imperative for the development of more effective tertiary prevention practices in real-life health care systems.

**Method:**

In this qualitative study, Finnish suicide attempt survivors’ experiences of and perspectives on mental health services were explored through a semi-structured interview. Participants were seven female and seven male service users interviewed 3–6 months after the index suicide attempt. A conventional content analysis of these service user interviews is presented.

**Results:**

Participants’ experiences of care ranged from helping to hindering recovery. Seven key aspects of services were described as helpful when present and hindering when absent. These included (1) meeting the service user as worthy of help, (2) supporting the exploration of personal meanings, (3) supporting the exploration of suicidality, (4) psychological continuity and predictability, (5) offering a responsive partnership in navigating recovery, (6) inviting service user involvement in medication decisions, and (7) accounting for service users’ relational context.

**Conclusions:**

Current health care services are inconsistent in meeting suicide attempt survivors’ subjective needs, leaving clear room for improvement in tertiary suicide prevention. To be perceived as meaningful by service users, services should strive to offer opportunities for both biomedical, psychological, and social interventions, with responsivity to individual needs and preferences. A focus on the social aspects of recovery (e.g., offering support to loved ones affected by the suicidal incident; facilitating peer support and social belonging) was most often found to be lacking in current services.

**Supplementary Information:**

The online version contains supplementary material available at 10.1186/s13033-022-00563-6.

## Background

A history of attempted suicide is the most significant predictor of suicide death [1], making suicide attempt survivors’ care a priority in suicide prevention. Tertiary prevention research aims at supporting improvement in practices. However, transforming research evidence into more effective real-life health care systems presents an ongoing challenge [2, 3]. Previous research has shown that suicide attempt survivors’ subjective experiences of care are related to, e.g., treatment outcome and future engagement with services [4, 5]. In-depth understanding of service user perspectives is thus needed to inform the development of approachable, high-quality services [6, 7].

Historically, Finland has pioneered suicide prevention efforts [8]. The Finnish National Suicide Prevention Project of 1986–1996 [9] was the first comprehensive, research-based suicide prevention program in the world. Its quantitative and qualitative results were published in over 100 articles [8, 10]. Prominent findings included a high incidence of untreated psychiatric disorders in individuals who had died by suicide and inadequacies in the treatment of suicide-related psychiatric disorders. National policies were implemented to improve identification rates and quality of treatment of these disorders.

The National Suicide Prevention Project was a success [9, 10]. Finland’s previously rising suicide rates began to decline in 1990 and have since halved [11]. However, the current age-standardized suicide rate of 13.4/100,000 remains above the average for high-income countries [12]. In 2020, the Finnish Ministry of Social Affairs and Health launched a new Mental Health Strategy and National Suicide Prevention Program for the decade 2020–2030 [13]. Measures detailed in the program include raising public awareness to reduce stigmatizing attitudes, restricting access to means of suicide, enhancing access to low-threshold crisis support and health care, supporting those bereaved by suicide, attending to substance-abuse-related suicide risk, improving responsible media coverage, developing EU legislation for suicide-related social media content, and strengthening research.

Since the completion of the National Suicide Prevention Project, Finnish suicide research has continued to yield results of value for tertiary prevention. The most recent publications include a clinical trial [14] and longitudinal observations on prospective study cohorts [15]. However, qualitative research efforts have been scarce, and suicide attempt survivors’ experiences of services remain unexplored.

### Finnish mental health services for suicide attempt survivors

Finland has universal health care that includes the promise of need-based psychiatric services for all residents, with recently published Current Care Guidelines [16] for suicide prevention and intervention after attempted suicide. However, treatment delays and the limited availability of evidence-based psychosocial interventions have been identified as barriers to appropriate care, with an ongoing national debate on possible solutions [17]. Despite prioritizing efforts, these barriers also affect individuals presenting with suicidal behavior [18].

While private-sector providers offer treatment options for those with private insurance or the ability to pay out of pocket, several non-governmental organizations (NGOs) supplement public health care with free-of-charge services. The NGO most prominently involved in suicide prevention, MIELI Mental Health Finland, provides crisis support services, a national crisis helpline, and the Attempted Suicide Short Intervention Program (ASSIP) [19]. ASSIP is a three-session manualized intervention for suicide attempt survivors, with follow-up letters and the possibility for crisis contact over the next 2 years. ASSIP is designed to be auxiliary to any health care interventions assessed as appropriate (i.e., treatment as usual) after a suicide attempt and is recommended as such in the Current Care Guidelines [16].

### Aims of the study

We investigated service users’ experiences of health care services after a recent suicide attempt. The present article focuses on service users’ experiences of services provided by the Finnish public health care system. These service users’ experiences of ASSIP will be presented elsewhere. We aimed for in-depth understanding of service users’ personal views on whether and how services had facilitated or could facilitate their recovery. Our data-driven definition of recovery emphasizes the present service users’ own understandings of its goals and process and resembles the concept of ‘psychological recovery’ proposed by Andresen et al. [20].

## Methods

This study applied an exploratory qualitative design in a naturalistic setting. Participants were suicide attempt survivors with recent experience of both health care services and the Attempted Suicide Short Intervention Program (ASSIP). Here, we report our findings on participants’ experiences with the health care system, i.e., “treatment as usual”. This includes experiences with, e.g., primary health care services, emergency services and psychiatric in-patient and out-patient services. Findings on participants’ experiences of ASSIP, provided by an NGO outside the health care system and designed as an adjunct to treatment as usual, are to be published separately.

Our primary data consist of in-depth service user interviews focusing on experiences of care. Additional data include written summaries of participants’ narratives of their index suicide attempt (documented as part of ASSIP). These summaries were reviewed in this study solely to enhance contextual understanding.

This study received ethical approval from the Helsinki University Hospital Ethics Committee. As per Finnish and EU regulations, participants were given a detailed description of the procedures for ensuring the confidentiality and protection of their personal data both during and after the study. All participants gave their written consent to use of their recorded interview and the written summary of their suicide attempt for the purposes of this study.

### Study recruitment

Participants were recruited through the MIELI Suicide Prevention Center (MIELI) in Helsinki. Eligible participants included all persons entering ASSIP at MIELI, excluding only those under age 18 and/or resident outside the Hospital District of Helsinki and Uusimaa. Through MIELI, ASSIP is available to Finnish-speaking adolescents and adults with a recent suicide attempt, excluding those whose suicide attempt occurred during a psychotic episode, those with a current substance abuse disorder serious enough to impede engagement with the intervention, and those with habitual serious self-harm. In the ASSIP context, a suicide attempt is defined as either a completed or interrupted action that, in the person’s own understanding, was aimed at taking their own life.

ASSIP therapists informed eligible clients of the study at beginning of the first ASSIP session and at the end of the last session asked for their consent to participate. Consent was confirmed by the interviewer at the end of the study interview.

### Participants

Of the 104 eligible service users informed of the study, 18 gave their initial consent and 14 participated in the research interview (one could not be interviewed due to COVID pandemic restrictions and three withdrew before the interview). The most common reason given for consenting was a desire to be of help in service development and/or increase public awareness of suicidal behavior. Reported reasons for non-consent included privacy concerns and/or an expectation that participation would be overwhelming. Participant characteristics are presented in Table 1. Participants represented diverse socio-demographic backgrounds and current life circumstances. Highest education varied from high school diploma to master’s degree. Thirteen participants were white, and one was of mixed ethnicity.

**Table 1:** Participant characteristics

	n	%
Registered sex		
Male	7	50
Female	7	50
Age		
18–29	5	36
30–45	4	29
46–59	3	21
60+	2	14
Current occupation		
Employed	7	50
Student	3	21
Pensioner	2	14
Unemployed	2	14
Living arrangement		
With spouse	4	29
With other adult family member(s)	3	21
Alone or with roommate	6	43
No fixed abode	1	7

The physical severity of the index suicide attempts ranged from requiring emergency medical intervention to interrupted with no physical injury (e.g., climbing to a height but deciding not to jump). Planned or used methods included intoxication (9), self-cutting (2), leaping from a height (2), motor vehicle collision (3) and electrocution (1); some participants combined means. In addition to the index attempt, eight participants reported at least one previous suicide attempt either in recent years or decades earlier. Seven had received psychiatric treatment in relation to suicidality before the current episode. During the current episode, all had experience of emergency services, 12 had received outpatient psychiatric services, four had been inpatients, and two had received psychotherapy.

The participants’ narratives of their suicide attempt showed diversity in the routes to suicidal action. Two participants reported psychological well-being well into middle age and attributed their suicide attempt solely or primarily to a specific current stressor (e.g., chronic pain due to a somatic condition). Three participants narrated a previous suicidal episode, followed by a lengthy period of well-being before the current episode. The majority of the participants narrated the suicidal process as having its roots in early childhood, many reporting traumatic life histories of early abuse and/or bereavement.

### Service user interviews

All participants took part in a semi-structured research interview conducted by the first author. The interviews took place at the MIELI Suicide Prevention Center 3–6 months after the index suicide attempt and 4–10 weeks after the last ASSIP session. Interviews lasted 45–120 min and were video recorded. Following the interview topic guide (see Additional file 1), experiences of ASSIP were investigated first, then those of any other services received by the participant. Participants were asked about aspects of services they perceived as helpful, unhelpful, or even hurtful, any surprising elements, suggestions for improvement, and their subjective assessment of whether each service received had been helpful to them. The primary focus was on the most recent suicidal episode, but accounts of previous episodes were explored when initiated by participants. While all the participants answered all the questions in the topic guide, the interviewer followed the participants’ narrative lead, and thus the order of the questions varied. A reflective journal was kept to document initial impressions, insights and questions elicited by each interview.

### Data analysis

We performed a conventional content analysis [21] of the interview data, since our aim was to describe the phenomenon under study (i.e., suicide attempt survivors’ perspectives on services) and this method allows data-driven insights to emerge from the data. Interviews were transcribed verbatim and read/listened to multiple times to enable immersion in the data. Data excerpts relevant to the research question (i.e., all meaning units in which participants expressed some kind of personal view on health care services) were then systematically identified and open-coded. Open-coded units of similar content were organized into clusters and the clusters tentatively conceptualized as themes. Data excerpts not yet belonging to established clusters/themes were reviewed in a cyclical process, resulting in the refinement of existing conceptualizations (incorporation of variations of closely related thematic content) and the formation of new clusters (when data did not fit with existing clusters/themes). A record was kept of the evolving coding and clustering of data and conceptualization of themes. The analytical process was led by the first author and reviewed and refined in data sessions with the fourth author. All authors contributed to refining the final themes and their wordings during the writing process.

## Results

The participants provided rich accounts of their personal experiences of and views on services they had received. In narrating their experiences, participants mentioned a variety of personally meaningful recovery goals, i.e., changes they wished for and/or understood to be a personal marker of “getting better”. Such goals included, for example, ridding oneself of the wish to die, not being overwhelmed by negative feelings, finding hope, (re)discovering an interest in working or the ability to work, and being able to meet the demands of daily life. Participants also spoke of a variety of recovery tasks, i.e., activities they understood as a route to achieving their personal goals. These tasks included, for example, learning to talk about what was bothering them, strengthening their sense of self-worth, getting traumatic experiences “off their chest”, finding the right medication, learning to manage recurrent suicidal impulses without acting on them, and finding or returning to meaningful activities and/or relationships.

When participants were asked about the helpfulness of services they had received, they seemed primarily to make these evaluations in relation to their personally meaningful recovery goals and tasks. Thus, services were found helpful when experienced as providing help in achieving personal recovery goals and/or working on personal recovery tasks and unhelpful or even hurtful when experienced as not supporting personal goals/tasks and/or promoting goals/tasks that the participant did not find personally meaningful.

Seven key themes emerged in the participants’ accounts of what helped or hindered their recovery. Themes 1–5 were found in all the participants’ accounts and themes 6–7 in most of them. We present these key themes as dimensions that incorporate the whole range of helpful to hindering experiences reported by participants.

### Key aspects of services perceived as helpful after a suicide attempt

**Fig. 1:**
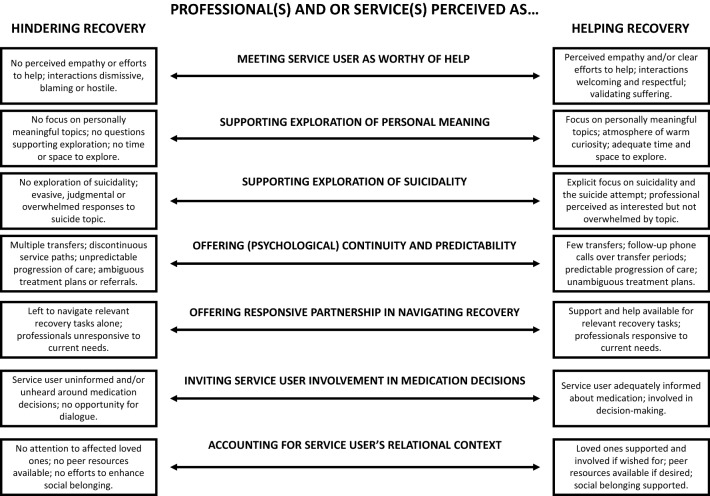
Key aspects of services perceived as helpful

The key aspects of services perceived by service users as helpful to recovery included *meeting the service user as worthy of help, supporting the exploration of personal meaning*, *supporting the exploration of suicidality*, *offering (psychological) continuity and predictability, offering a responsive partnership in navigating recovery*, *inviting service user involvement in medication decisions* and *accounting for service users’ relational context* (see Fig. 1).

#### Meeting the service user as worthy of help

This theme refers to how professionals were perceived to communicate that the service user was (or was not) deserving of help. The participants most often described the professionals they had encountered as well-meaning. They spoke appreciatively of “understanding”, “empathetic” or “decent” professionals expressing genuine concern, working to arrange for their continued care, and giving them information about their options and encouragement about the possibility of recovery. Such actions were experienced as validating service users’ worth as human beings, reducing shame and evoking hopefulness. Participants who had hesitated to disclose their suicide attempt cited professionals’ empathetic style as making disclosure possible and/or worthwhile.

Participants mostly described professionals’ actions as understandable (e.g., caused by an overwhelming workload) even when they felt hurt or disappointed in some way by those actions. There were, however, exceptions. Some participants read staff unresponsiveness to their individual circumstances as a cue that their treatment was being performed “for the organization, not for me” or “as a routine”, resulting in a feeling of being dismissed or not deemed worthy of individualized care. Many also reported of a professional acting in ways that felt intentionally punitive and/or blaming, such as aggressively commanding “a grown-up” to “stop playing around”. These incidents were described as hurtful, but they did not seem to hinder participants from having subsequent good experiences with other professionals. However, several participants described learning to fear and/or avoid a specific treatment context (most often the emergency room) due to hostile or humiliating interactions with staff that they had experienced themselves or witnessed peers experiencing. Some reported this as a personal barrier to care and as accelerating self-harm behaviors.

#### Supporting the exploration of personal meaning

This theme included accounts of professionals’ perceived support (or lack thereof) in the exploration of themes and experiences that the participants found meaningful in relation to their suffering, including relationship issues, unresolved life experiences and questions of identity. Such exploration was desired by all participants and cited by many as the most important aspect of care. However, several participants felt that issues such as medication, diagnoses, sick leave, and/or management of anxiety had been over-emphasized in their care, while little or no attention was paid to understanding the roots of their subjectively experienced suffering. Participants expressed wishes of “[professionals] really getting to know me”, “going deeper”, “focusing on root causes” and “more therapy-type sessions”.

The participants made it clear that although they were motivated to explore difficult topics, they needed help in doing so. Several participants emphasized that without the support of questions they would be or had been unable to express themselves. One participant stated, “if they didn’t ask me anything, I wouldn’t say anything” and another reported sitting in anxious silence and eventually dropping out of appointments in which professionals “seemed to expect I could just open up” with very little help from questions.

When exploring personally meaningful topics with an engaged professional, the participants described gaining new insights and feeling less shame and more compassion for themselves. However, opportunities for such exploration seemed to be inconsistent across services. While some participants reported appreciatively on such exploration with their psychologist or nurse, others felt there had been no room for this in their health care contacts. This seemed to lead several participants to wish for psychotherapy, which they expected would offer them an opportunity for the kind of joint exploration they longed for. In fact, this opportunity seemed to represent the most significant line of hope for several participants. In some, these hopes had a desperate tone, since either costs or difficulties in finding a service provider made psychotherapy seem like it was “not an option” or “just a pipe dream”.

#### Supporting the exploration of suicidality

This theme comprised accounts of professionals’ perceived support (or lack thereof) in the exploration of the participants’ suicidality. All participants viewed careful examination of the suicide attempt as important or even crucial for formulating meaningful recovery goals or treatment plans. However, many felt that there had been little or no opportunity for this in their health care. Some participants reported that they had only been asked about suicidality through standard questionnaires, and that their answers were not subsequently discussed with any professional, one participant stating, “I felt like I was filling in forms all the time—I have no idea where they went”. Another reported a nurse in a psychiatric ward telling her not to talk about her suicide attempt, as “it’s time to move on now”. Several others also felt that the topic of suicidality seemed to be avoided by professionals, sometimes creating a severe obstacle to collaboration. The exploration of suicidality was thus raised as an issue separate from (although parallel to) the exploration of personally meaningful topics in general.

Participants also reflected on their personal struggle with the topic of suicidality, acknowledging it as “difficult to talk about” and “not something you want to repeat every time to a new professional”. Some had hesitated over the disclosure of suicidal intent or a suicide attempt due to hopelessness about treatment and wanting to retain the option of completed suicide. One participant reported that despite her hesitation she would have disclosed her intent before the attempt, had she been asked directly about suicidality by her psychiatrist. She reported being surprised at not being asked.

Several participants reported hesitating over discussing suicide topics due to worry about the effects on professionals. Many had anticipated or perceived professionals to be emotionally burdened by their accounts of suffering and/or suicidality, one participant stating, “I kind of feel bad going through all this with [professionals], like, how can they take it—I’m making them feel bad, too”. On the other hand, participants expressed appreciation for situations in which they felt talking about suicidality was “allowed” and professionals did not become, for example, “overwhelmed”, “either overly concerned or withdrawn” or “judgmental” around this topic.

#### Offering (psychological) continuity and predictability

This theme included accounts of the perceived (dis)continuity and/or (un)predictability of services. Most participants expressed a wish for more continuity of treatment. Many had felt demoralized by being repeatedly transferred from one professional to another. Several stated that starting with a new professional felt like “going back to the beginning” and disrupted their progress. Some constantly feared news of another transfer, having previously lost a meaningful treatment relationship due to, e.g., staff changes.

Participants reported feeling that to avoid being prematurely discharged they needed to be rather proactive in their engagement with services. This led to much unease, as many recognized that hopelessness and/or fears of being burdensome could dissuade them from using services. Follow-up contact with suicide attempt survivors was a common suggestion for service improvement, with several participants emphasizing the importance of professionals checking on the outcomes of emergency room referrals.

Participants’ sense of service continuity was sometimes challenged by confusing or unclear treatment plans. Many were pleasantly surprised at receiving their first psychiatric appointment within just days of referral. However, they also reported professionals emphasizing that treatment would be of limited (but unspecified) duration, leaving them in uncertainty about the availability of care in the near future. Some reported being left in confusion during a transfer period about whether, how or where their treatment might continue. On the other hand, a supportive phone call during a transfer period could greatly improve participants’ satisfaction with the continuity of their treatment paths. The participants’ emphasis was thus on the psychological or experienced continuity of care rather than the number of transfers per se*.*

Some participants’ sense of psychological continuity was further undermined by cognitive dysfunction during the most acute phase of their suicidal crisis. They had noticed with frustration that even helpful interactions and insights soon became unretrievable from memory during this phase. Several participants wished for more written notes (on both practical information and insights during treatment sessions) and text message reminders.

#### Offering a responsive partnership in navigating recovery

This theme included participants’ perceptions of the responsivity (or lack thereof) of professionals to their individual circumstances, needs and preferences in the recovery process and the collaboration offered by professionals in navigating it. While they wished for therapeutic conversation, the participants rejected an exclusive focus on this or any other form of intervention. Instead, they wished for need-based support to be available for a variety of personally meaningful recovery tasks. These included, e.g., arranging for basic needs (e.g., applying for benefits, finding an apartment), organizing meaningful day-to-day activities (especially during sick leave) and finding peer support. In tackling current issues, participants emphasized their wish for partnership or collaboration with, rather than simple direction from, professionals. Such collaborative interactions had been experienced by most participants at least some of the time. These experiences were described as, e.g., “empowering”, hope-evoking and encouraging further engagement with services.

However, collaboration or responsivity to service users’ expressed needs was not a given. Some participants felt that professionals’ views on relevant recovery tasks had differed widely from their own and that reconciling these differences had proven difficult. One participant felt her hopelessness was currently largely due to the interruption of her studies, making resuming these studies her prioritized recovery task. Completing this task would have required making a phone call to the school, a “simple” task greatly complicated by her anxiety. Thus, she wished that “someone would [make the call] with me, since I can’t do it alone”. However, she felt that when she spoke about this issue, “[professionals] told me that kind of stuff is easy to fix”. Yet she felt no help was offered in fixing it, thereby exacerbating her hopelessness.

Several participants perceived the organizational context (policies, workloads etc.) as restricting professionals’ responsivity to service users’ individual circumstances. This seemed to result in experiences of objectification, with some participants describing treatment as “something that’s done *to* me” or as moving along an “assembly line” rather than a collaborative process. Doctors’ (including psychiatrists’) roles were often perceived as disappointingly restricted to such topics as diagnoses, medication, and sick leave. Organizational protocols, culture or constraints were also the perceived cause of many unsatisfactory interactions with other professionals. One participant reported attempting to initiate dialogue on treatment tasks and goals by asking his psychologist about “the point of these sessions”. The reply, “you are entitled to these specialized psychiatric services”, seemed to him confirmation that his treatment was performed primarily as an organizational routine, with individual needs and recovery tasks deemed irrelevant.

#### Inviting service user involvement in medication decisions

This theme included accounts of participants’ experiences with psychotropic medication and professionals’ perceived efforts (or failures) to collaboratively engage participants in dialogue about it. Thirteen participants reported receiving some kind of psychotropic medication in relation to their recent suicidal crisis. Twelve reported having experienced adverse side-effects and/or withdrawal symptoms (e.g., extreme fatigue, “feeling drunk”, nausea and heart palpitations). Two were certain of the helpful effects of medication and two others assumed this, reporting that medication “can’t be ruled out as a cause for feeling better” or “I don’t remember how I felt without it, but I assume it’s helpful”. The remaining nine had to date no personal experience of the benefits of medication. However, almost all participants reported being at least somewhat hopeful about the potential of medication being helpful, and even those who were not hopeful, reported compliance.

In fact, several participants stated that medication is an important—“even the most important”—element in treatment, despite having no personal experience of its helpful effects. However, even participants with high hopes for medication expressed dismay at situations in which it seemed the primary focus of their care. As one participant stated:

“Even though [medication] is the most vital part of treatment, it felt a bit much once when I came in and the first thing I’m asked is ‘how’s the medication, have you taken it?’. I mean, I felt like they could at least ask how I’m doing and not the meds [small laugh]. But that’s just me, I mean the meds are an important part of it and that’s how it should be.”

Most participants also expressed frustration in receiving little or even no information on the medication prescribed for them, the difficulty of “finding the right drug”, and/or doctors being “unable to explain how or why [the medicine] should work”. Many participants expressed a wish for genuine dialogue with their doctor about medication, possible adjustments to it and/or its eventual termination.

#### Accounting for service users’ relational context

This theme included participants’ perceptions of professionals accounting (or not) for their social and relationship context. All participants with a spouse or involved adult children expressed concern about their family members being affected by the suicidal incident and receiving too little or no support. They wished for “a system for this” and that it would not be left up to family members and/or service users alone to decide if, when and how they might need support or want to join the treatment process. Some participants also reported conflicts in close relationships that contributed to their suffering but remained unaddressed in their treatment.

Most participants described support from family members and/or friends’ as a valuable resource in their recovery. However, this resource did not seem to receive much attention in their health care contacts. In addition to loved ones not being offered support and not being invited to join treatment processes, participants expressed dismay at experiences such as having no private place to go with visitors during an inpatient stay or being discharged from the emergency room without a family member being informed, despite requests both from themselves and family members. Some participants, however, considered it important that family members were *not* involved in their treatment.

Those with scarce natural networks called for their lack of close relationships or thwarted social belonging (e.g., during sick leave) to be better taken into account in treatment planning and practices, including more active checking-in by professionals “to keep track that I’m alive”. They also expressed appreciation for efforts to provide “human contact” through services even if they were unhappy with other aspects of their health care contacts.

Some participants felt group interventions better suited “less grave situations and more outgoing people” or feared their own reactions to peers’ difficult emotions, while others had found or expected to find both formal and informal peer interactions highly valuable. Some participants emphasized the importance of both peer relationships and written narratives by recovered peers as resources providing experiences of social belonging, hope and destigmatization. However, they had found professionals to be mostly unaware of such resources and unable to give guidance on finding them even when asked.

## Discussion

This article reports on service user experiences of health care services after a recent suicide attempt, focusing on both helpful and hindering aspects of care. All the participants had received the Attempted Suicide Short Intervention Program (ASSIP) [19], provided by a non-governmental organization outside the health care system and designed as an adjunct to treatment as usual. Findings on users’ experiences of ASSIP will be published elsewhere and are discussed here only briefly as context for the present findings.

A recent Finnish randomized controlled trial [14] comparing ASSIP and crisis counseling as usual (CC) as adjuncts to treatment as usual provided by the health care system found a non-significant difference in effectiveness between these interventions in preventing repeat suicide attempts. The high re-attempt rate in both groups (29.2% for ASSIP and 35.2% for CC at 2-year follow-up) indicated an urgent need for the development of the whole service system. We believe the present in-depth qualitative exploration of recent service user experiences has provided information useful for improving services.

As in earlier studies, e.g., [22, 23], the present service users had ample experience of both helpful and hindering (or even hurtful) interactions with services. Key aspects of services perceived as helpful in their pursuit of recovery included *meeting the service user as worthy of help, supporting the exploration of personal meaning*, *supporting the exploration of suicidality*, *(psychological) continuity and predictability*, *offering a responsive partnership in navigating recovery*, *inviting service user involvement in medication decisions* and *accounting for service users’ relational context.*

Our findings are both congruent with and complement previous research. Irrespective of context, suicide attempt survivors wish for collaborative professionals and continuity of care, including more follow-up efforts and fewer transfers during treatment processes, e.g., [5, 23, 24]. More attention to peer and natural network resources have also been requested by suicide attempt survivors in previous studies, e.g., [22, 25]. Service users also frequently perceive some professionals as unprepared to discuss suicidality, e.g., [22, 25, 26]. The service users in this study emphasized the importance of early and consistent opportunities for both the therapeutic exploration of meaningful topics and biomedical interventions to alleviate suffering, the one not being seen as a substitute for the other. Similar appreciative and critical views on psychotropic medication have also been reported in previous studies, e.g., [22].

Suicide attempt survivors’ appraisals of helpful aspects of care mostly coincide with those presented by other psychiatric service users, e.g., [27]. However, careful exploration of the suicidal act may be considered as a need specific to this service user population. While not systematically highlighted in previous qualitative studies, this need was emphasized by the present participants. In short, these service users join those in earlier studies who have called for patient-centered care with need-based opportunities for a variety of interventions, e.g., [23, 24].

### Reflections on the Medical Model

The frustrations users report with current services may be seen as reflecting Medical Model-related issues previously addressed in the literature [6, 7, 28–31]. The Medical Model is the paradigm favored by Western modern medicine. Despite controversy, it also dominates both research and practice in the fields of psychiatry and suicidology. In the Medical Model, suicidal behavior is understood as symptomatic of an underlying illness or disorder of the individual (e.g., depression) for which curative or symptom-reducing treatment is seen as the primary route to preventing further suicidal behavior. Acceptable interventions posit targeting a specific cause of this illness or disorder with an effective specific ingredient, whether biological (e.g., psychotropic medication targeting a neurochemical imbalance) or psychological (e.g., a specified therapeutic intervention targeting suicidal cognitions). As cures are understood to be disorder-specific, standardized assessment methods (e.g., symptom inventories) are preferred to ensure accurate diagnosis. With mounting quantitative evidence [4], such common factors as the therapeutic alliance are increasingly recognized as relevant, but their value is seen as indirect or instrumental (e.g., enhancing adherence to treatments delivering specific ingredients) rather than healing per se.

While the Medical Model may be credited with many advances in modern psychiatry and suicide prevention, its challenges in alone informing effective responses to mental health issues in general and suicidal behavior in particular have been repeatedly addressed in the literature (e.g. [6, 7, 27, 28]). The present findings may be seen as reflecting these challenges. The Finnish Current Care Guidelines [16] for suicide prevention and intervention after attempted suicide acknowledge the existence of alternative models of suicidal behavior, i.e., that suicidal behavior may be understood as at least partly independent of any illness or disorder. However, these guidelines rest firmly on the Medical Model, as do the health care practices informed by them. In their appraisal of these practices, the present service users echoed criticisms of the Medical Model in reporting frustration with what they perceived as an overly individual focus in care, an over-emphasis on medication, diagnoses and standardized procedures, an inadequate focus on the underlying interpersonal or social causes of suicidality, and treatment discontinuity caused by the structuring of services. These practices were often perceived as objectifying and contributing to a sense of not being seen or valued as one’s unique self. On the other hand, when professionals’ general stance was perceived as empathetic and collaborative, Medical Model-informed intervention contents (e.g., psychotropic medication, referral to specialized services) were often highly valued by the participants.

Interestingly, many service users seemed to be caught up in a personal debate about the most efficacious model of responding to suicidality. In their accounts, they argued consecutively for the primacy of medication and the primacy of psychological or social interventions in suicide prevention. These service user reflections presented an interesting parallel to the controversy and debate among professionals, communicating a similar co-existence (rather than achieved integration) of different paradigms. Echoing Engel’s [32] classic proposition of a biopsychosocial model for the treatment of mental health issues, most of the service users offered framings of suicidality as *both* (1) symptomatic of an illness with biological causes and thus curable with medication, (2) expressive of psychological vulnerabilities and thus suitable for psychological interventions, *and* (3) as rooted in their social context and thus best alleviated by interventions targeting their relationship with this context.

The Finnish Current Care Guidelines [16] also state that biological, psychological, and social factors all contribute to the pathway to suicidal behavior. However, social factors seem to be largely overlooked in current health care practices, perhaps due to their awkward fit with the Medical Model (see also: [33]). In the present study, all the participants had been offered biological remedies and at least some form of psychological support or intervention, as laid down in the Finnish Current Care Guidelines. But while these guidelines cite, e.g., community support as a protective factor, they do not suggest possible interventions targeted at social or interpersonal aspects of recovery. In keeping with these non-specific guidelines, few service users in the current study reported receiving support focusing on the social aspects of recovery.

The present service users seemed, however, to find such recovery tasks highly relevant. They called attention to their social context in expressing worry about affected loved ones or sorrow over their lack of close relationships. Those who had been assigned sick leave often described being thrown further off balance by loss of the social roles associated with work or study and needing (but rarely receiving) help in adjusting to, or compensating for, this. Many saw relationships with peers as potentially highly meaningful and wished for (but rarely received) help in finding such resources. The conclusion Kerkhof ([10], p 63) reached two decades ago in an evaluation of the Finnish Suicide Prevention Program has not yet lost its relevance: “[t]here still appears to be a gap between medical paradigms and sociocultural paradigms in understanding and preventing suicidal behavior”. Our results, like those of earlier qualitative studies, underline the importance of finding ways to close this gap in order to provide effective, need-based interventions for those at greatest risk of suicide.

### Ethical considerations

While the value of service user participation in suicide research is evident, study designs require careful ethical consideration to prevent any adverse consequences for participants in this highly vulnerable population, e.g. [34]. Hence the present effort to address key ethical issues included procedures to ensure genuinely voluntary participation, safety in the event of heightened distress during or after the interviews, and protection of the participants’ data and identity. These procedures seemed to ensure safe and meaningful participation: all the service users reported satisfaction with their participation, even when they acknowledged feeling somewhat fatigued after the interview. Several participants described participation as a deeply meaningful experience and many spontaneously expressed their willingness to further participate in similar efforts. One participant described the experience:

“I find it really valuable to be able to put these experiences in words and know that someone is interested in this side of things…the view of someone navigating these processes and their perspective, in a deep sense, on the treatment they have received…I mean, I’ve filled in feedback forms in the past, but they feel kind of faceless…When I was considering participating, I knew I had stories to tell, this is not my first time around, and it feels [valuable] to be able to share my perspective.”

Ethical concerns include recognition of both researcher positioning and procedures enhancing validity [35]. This study was inspired by the first author’s wish to understand the experience of those using the psychiatric services she was also engaged in providing. This positioning may be seen as both an advantage and a threat to validity. While the first author’s personal engagement with the target service system allowed for a deeper contextual understanding of the participants’ accounts, it may also have presented risks through, e.g., preconception bias. The validity-enhancing procedures included a reflective journal (documenting a genuine learning process, including surprises, during the data collection and analysis), data sessions and discussions with other members of the research group, and dialogues with several peer audiences to invite multivoiced challenges to the emerging analyses.

### Strengths and limitations

The service users participating in this study were diverse in age, sex, socioeconomic status, previous service use and history of suicidal action. However, only a minority of those eligible decided to participate. While the uptake rate may be considered good for a qualitative study requiring such deep participant engagement, it is important to note the possibility of self-selection bias in the sample when interpreting the results. Service users with more resources and further along in their recovery are likely over-represented in this sample. The scarcity of minority representation in the sample limits the usefulness of these findings for understanding service experiences in minority groups vulnerable to both negative service experiences and suicide. Future studies could also include thus far understudied groups such as persons for whom a suicide attempt has resulted in permanent physical disability. Themes identified in this study may form a useful starting point (e.g., in brief questionnaire form) for a quantitative investigation of service experiences in a representative sample of service users.

We plan to report findings on participants’ experiences of health care services and ASSIP in separate publications to allow for a more detailed exploration and discussion of each. However, service users’ experiences of ASSIP have likely affected their appraisals of encounters in the health care system, and vice versa. ASSIP seemed to benchmark some desirable aspects of care, which may have resulted in greater service user frustration with other services. In the first 1–2 months following ASSIP, psychiatric services seemed to fail as often as they succeeded in supporting service users’ continued work on recovery tasks that they had identified as personally relevant during ASSIP. Service users left without such support experienced this discontinuance as undermining the gains they had made in ASSIP, while those receiving such support felt they were further building on these gains.

## Conclusion

In this study, we sought in-depth understanding of suicide attempt survivors’ perspectives on health care services after a suicide attempt. We believe our findings are useful for both clinicians, service developers and policy makers. In line with previous research, service users reported that being met with empathy and respect fostered a sense of hope, self-worth and belonging, while hostile or dismissive staff attitudes created barriers to care and even accelerated self-harming behaviors. Adequate predictability and continuity of services was perceived as crucial for both making and retaining recovery gains. Service users called for the need-based availability of both (bio)medical remedies, psychological interventions (including an explicit, but not exclusive, focus on exploring suicidality), and interventions targeting their relational context and sense of social belonging. The responsiveness of services to individual needs and preferences was described as key, with service users emphasizing that one valued intervention modality (e.g., psychotropic medication) cannot substitute for another (e.g., therapeutic conversation). Interventions targeting social aspects of recovery (e.g., attention to affected loved ones; facilitation of peer support and social belonging) were most often found to be lacking in current services.

## Supplementary Information

**Additional file 1. ** Interview topic guide. Translated from original Finnish.

## Data Availability

The original qualitative data is not available due to participants’ right to privacy.

## Abbreviations

ASSIP: Attempted Suicide Short Intervention Program
CC: Crisis counseling
NGO: Non-governmental organization
MIELI: MIELI Mental Health Finland Suicide Prevention Center
