# Supplementary material for: How do health care services help and hinder recovery after a suicide attempt? A qualitative analysis of Finnish service user perspectives
Source: Int J Ment Health Syst. 2022 Nov 16;16:52. doi: 10.1186/s13033-022-00563-6 (PMC9670548; doi:10.1186/s13033-022-00563-6)
Supplement: Supplementary file 1 — Additional file 1. Interview topic guide. Translated from original Finnish. [file 13033_2022_563_MOESM1_ESM.docx]

**Additional file 1**

**Interview Topic Guide**

1. What made you decide to participate in this interview?
2. How did you become a client of ASSIP?
   1. How did you find out about ASSIP?
   2. What made you decide to participate?
3. What has participating in ASSIP been like for you?
   1. Has any aspect of ASSIP surprised you? If so, what and how?
   2. What aspect of ASSIP, if any, has been most helpful for you?
   3. Have you found any aspect of ASSIP unhelpful or even hurtful in some way? If so, what and how?
   4. Has ASSIP helped you to move forward?
   5. What, if anything, could have been done better or differently?
4. What other services related to your suicidal crisis have you received, either before or since the suicide attempt?
5. What has participating in (or receiving) this service been like for you? *[Asked separately for each service mentioned by the participant.]*
   1. Has any aspect of this service surprised you? If so, what and how?
   2. What aspect of this service, if any, have you found most helpful?
   3. Has any aspect of this service been unhelpful or even hurtful in some way? If so, what and how?
   4. Has this service helped you to move forward?
   5. What could have been done better or differently?
6. What, if anything, has been expected of you as a user of these services?
   1. How have you felt about these expectations?
7. What hopes or expectations do you have regarding services now or in the near future?
8. What aspects of care do you consider most important, if you think about helping a suicidal person or a suicide attempt survivor in general?
9. What has help from non-professionals meant for you during your suicidal crisis?
10. Is there anything else you would like to say about your experiences?
11. What has participating in this interview been like for you?
